# Advances in PET and MRI imaging of tumor hypoxia

**DOI:** 10.3389/fmed.2023.1055062

**Published:** 2023-02-09

**Authors:** Pierrick Gouel, Pierre Decazes, Pierre Vera, Isabelle Gardin, Sébastien Thureau, Pierre Bohn

**Affiliations:** ^1^Département d’Imagerie, Centre Henri Becquerel, Rouen, France; ^2^QuantIF-LITIS, EA 4108, IRIB, Université de Rouen, Rouen, France; ^3^Département de Radiothérapie, Centre Henri Becquerel, Rouen, France

**Keywords:** hypoxia, cancer, [^18^F]-FMISO, [^18^F]-FAZA, [^64^Cu]-ATSM, OE-MRI

## Abstract

Tumor hypoxia is a complex and evolving phenomenon both in time and space. Molecular imaging allows to approach these variations, but the tracers used have their own limitations. PET imaging has the disadvantage of low resolution and must take into account molecular biodistribution, but has the advantage of high targeting accuracy. The relationship between the signal in MRI imaging and oxygen is complex but hopefully it would lead to the detection of truly oxygen-depleted tissue. Different ways of imaging hypoxia are discussed in this review, with nuclear medicine tracers such as [^18^F]-FMISO, [^18^F]-FAZA, or [^64^Cu]-ATSM but also with MRI techniques such as perfusion imaging, diffusion MRI or oxygen-enhanced MRI. Hypoxia is a pejorative factor regarding aggressiveness, tumor dissemination and resistance to treatments. Therefore, having accurate tools is particularly important.

## 1. Introduction

Hypoxia is characterized by insufficient oxygenation of certain tissues or the entire body. This hypoxia results from an imbalance between oxygen supply and tissue consumption. Hypoxic tissues are a hallmark of advanced solid tumors and can constitute up to 60% of the tumor mass. Importantly, the distribution of hypoxic tissue in the tumor is heterogeneous and variable over time (1). However, the partial oxygen pressures found in tumor tissue adjacent to healthy tissue are similar because of the same distance from a nutritive blood vessel (2).

The role of hypoxia in the increase of radioresistance was demonstrated at the beginning of the 20th century by Schwarz (3), then in tumor radioresistance by Mottram (4, 5). In 1953, the crucial role of oxygen in the response to radiotherapy was fully demonstrated (6). Hypoxia allows a selection of more aggressive tumor clones on the one hand and, on the other hand the entry into dormancy of other cells (2, 7, 8). Thus, this phenomenon can lead to a decrease in response to radiotherapy and chemotherapy, an increase in the risk of metastasis and a poor prognosis for the patient. In addition, oxygen is a powerful radiosensitizer with an amplifying effect on the ionizing radiation. This amplifying effect by oxygen is greater than that obtained by the various radiosensitizing chemotherapies currently used. Indeed, ionizing radiations have a lethal effect on tumor cells, essentially by double strand breakage. These breaks can be obtained by the direct effect of radiation (energy deposition on the DNA double helix) and more frequently by indirect effect. This indirect effect is obtained by the creation of free radicals secondary to the radiolysis of water, which are stabilized in the presence of oxygen (9).

The reference method for the assessment of tumor hypoxia is the measurement of the partial pressure of oxygen in the tissue. This measurement is conducted invasively by polarographic histography of pO_2_ via implantation of an Eppendorf electrode in the tissue of interest. The first trials were conducted in the early 1990s by Höckel and Vaupel (10–12). The main disadvantages of the Eppendorf electrode are the degradation of the tissue since it must be brought into different tissue territories and the fact that only one measurement is possible per territory. This technique is not very applicable in clinical routine because of its invasive character and limited to easily accessible tumors. Moreover, the Eppendorf electrode does not allow to distinguish between necrotic and viable anoxic tissues. Non-invasive imaging methods have progressively been developed in nuclear medicine and radiology (13–15).

Thus, in radiology, it is possible to visualize in functional magnetic resonance imaging (fMRI) by blood oxygen level dependent (BOLD) effect showing the increase in concentration in oxygenated hemoglobin (16). Indeed, the increase concentration of oxygenated blood in the capillaries increases the T2* relaxation time of the protons and vice versa. It is also possible to measure directly the concentration of molecular oxygen in the tissues by electron paramagnetic resonance (EPR), but this method remains experimental (17). Also, other methods of exploration by MRI are possible and discussed in this review, such as the apparent diffusion coefficient, diffusion MRI, T1 and T2 sequences, quantitative MRI, based on the measurement of intrinsic parameters inspired by NMR relaxometry methods. DCE-MRI (injection of gadolinium contrast medium) provides a quantitative compartmental analysis to evaluate permeability and perfusion parameters and to identify hypoxic territories.

In nuclear medicine, tissue hypoxia can be assessed by nitroimidazole tracers ([^18^F]-FMISO, [^18^F]-FAZA, [^18^F]-HX4) or [*Cu]-Cu-ATSM. It is possible to study indirectly the variations of intracellular pH as a consequence of hypoxia by [^89^Zr]-girentuximab for example.

The purpose of this review is to provide an update on tumor hypoxia imaging techniques. These include low contrast PET imaging and emerging MRI imaging. The knowledge of hypoxic territories is crucial for the adaptation of radiotherapy or the resistance of tumors to chemotherapy.

## 2. Materials and methods

We searched the Pubmed^®^ database using the following both as text and as MeSH terms: “Hypox* + PET + Imaging + Cancer” over a period from 1990 to 2021. No language restriction was applied to the search. The systematic literature search returned 730 article abstracts.

Search results were judged for relevance using the title, abstract, and full text for inclusion in the analysis. Three researchers (one physician, one pharmacist and one scientist) performed the research and the critical analysis of the articles. We were able to select 178 articles whose abstracts were all read independently by the 3 researchers. We thus selected 118 articles, all of which were read extensively to select 90 works. We proceeded in the same way for the section on MRI with the following key search: “Hypox* + MRI + Imaging + Cancer” from 1990 to 2021, we found 836 articles. The same scoring system was applied to select 58 of them.

The systematic selection allowed us to find 148 works for this literature review.

## 3. Tumor hypoxia

### 3.1. Physiopathology

Tumor hypoxia results from an imbalance between the rate of cellular oxygen consumption and oxygen delivery to the cells. Hypoxia can be caused by a defect in tissue perfusion, a deficit in the diffusion of oxygen to the cells, or even a lack of hemoglobin following anemia. Hypoxia related to a perfusion defect is called acute hypoxia. The blood flow in the tissues is inadequate to the oxygen consumption of the cells composing these tissues. Thus, in hypoxic tumors, the newly formed capillaries are structurally and functionally abnormal. The vascular network is disorganized, with elongated and tortuous shapes, the endothelium is incomplete, in some places lacking receptors. These vessels do not allow regulation of blood flow, which can cause pauses in the movement of red blood cells. However, this ischemic hypoxia is often transient and contributes to the spatial and temporal heterogeneity of the distribution of hypoxic tumor tissue. While hypoxia related to a defect of oxygen diffusion is called chronic hypoxia. This type of hypoxia is caused by an increase in the diffusion distances of oxygen because of tumor expansion. This leads to an insufficient supply of oxygen to cells that are more than 70 μm away from the blood vessels. Chronic hypoxia can also be caused by a change in the diffusion geometry. In the blood network, there are movements and countercurrent flow movements in the same blood supply irrigation territory (7).

Thomlinson et al. have shown that hypoxic tissue is a feature of most solid tumors and can make up to 60% of the tumor mass (18). Paradoxically, tumor hypoxia is not specific to large tumors but is found in early stages, even in small (<1 mm^3^), poorly vascularized tumors. The newly formed tumor vessels are immature, branched and permeable, inefficiently perfusing the tumors (7).

Chronic and acute hypoxia can thus coexist in tumors, even small ones, with physiological consequences that need to be considered in the therapeutic approach to tumors. This is a dynamic process called “cyclic hypoxia.”

Finally, another type of hypoxia may be caused by a decrease in the oxygen transport capacity of the blood due to anemia caused by the tumor itself or following chemotherapy. Below a hemoglobin level of 10 g/dl the oxygen supply is compromised. The same type of hypoxia is found when hemoglobin is no longer able to transport oxygen due to poisoning (carboxyhemoglobin, cyanohemoglobin).

### 3.2. Consequences of hypoxia

We have to distinguish acute hypoxia versus chronic hypoxia. If the acute hypoxia is transient, the cells are in a state of apnea and then recover without damage, but if this hypoxia lasts long enough the exposed cells die. Chronic hypoxia affects cells in a slightly different way. Tumor cells may die following the p53 pathway or, on the opposite, chronic hypoxia may promote certain cell clones that are resistant to chemotherapy or radiotherapy (8). This resistance may be mediated by HIF-1α (hypoxia inducible factor). When the oxygen concentration decreases in cells, HIF-1α is no longer degraded and accumulates in the cell nucleus. It then binds to HIF-1β and recognizes the HRE (hypoxia responsive element) DNA sequence.

Chronic hypoxia (pO_2_ < 7 mmHg) also promotes tumor propagation by adapting cells to the limitation of nutrient supply, or by allowing them to leave their hostile environment by facilitating their proliferation, local invasion and metastatic dissemination. Indeed, about 30 genes are located downstream of the HRE sequence. They allow the expression of proteins involved in the improvement of oxygen supply [Vascular Endothelial Growth Factors (VEGFs); inducible nitric oxide synthase (iNOS)], in energy saving [Glucose Transporters (GLUTs); glycolytic enzymes], in cell survival and proliferation [Epidermal Growth Factors (EGFs); Insulin-like Growth Factor-2 (IGF-2); Transforming Growth Factor-beta (TGF-b)] and in the fight against acidification of the cell environment [Carbonic Anhydrase 9 (CA-IX)].

Finally, when pO_2_ < 0.7 mmHg in tissues, an increase in the number of cell mutations is observed. Hypoxia exerts a very strong selection pressure and only the fittest and probably the most aggressive cell variants survive due to the intense expression of genes located downstream of the HRE sequence. Furthermore, this selection pressure from the environment can generate intratumor heterogeneity with one clone dominating the others in one part of the tumor, while another dominates in another part. So, the cells are more and more adapted and aggressive until the dissemination of these clones in the whole organism.

All these considerations established tumor hypoxia as a negative prognostic marker in almost all solid tumors and the necessity to have precise and efficient imaging tools (19).

### 3.3. Targets for hypoxia imaging

#### 3.3.1. Glucose metabolism

Clavo et al. have suggested that the most widely used PET-radiotracer, [^18^F]-fluorodeoxyglucose ([^18^F]-FDG), could also be used as a surrogate for hypoxia imaging since glucose metabolism is activated via HIF1α under hypoxic conditions (20). In 2005, Zhao et al showed that the [^18^F]-FDG uptake pattern as well as the expression of glucose transport proteins and hexokinase are related to the presence of HIF-1α by use of autoradiography and immunostaining (21).

Tumor hyperglycolysis due to upregulation of GLUT glucose transporters and glycolytic enzymes explains its interest as a surrogate marker of hypoxia. The [^18^F]-Fluoro-D-Glucose ([^18^F]-FDG) used in PET imaging, as a tracer of the carbohydrate metabolism of cells, has been proposed in the literature to visualize tumor hypoxia. [^18^F]-FDG would thus allow an indirect measurement of cellular hypoxia.

At the same time, a significant decrease in pO_2_ may lead to a state of tumor “dormancy,” characterized by a dynamic balance between the multiplication of sufficiently oxygenated tumor cells and the death of hypoxic tumor cells. In most cases, these tumors regress spontaneously, especially in response to immune system attacks. However, in rare cases, hypoxia promotes the emergence of tumor cells capable of producing energy by anaerobic glycolysis by recycling lactate, which in turn can positively regulate GLUT1 expression and thus modulate [^18^F]-FDG uptake.

[^18^F]-FDG is the most available tracer in PET/CT imaging. As early as 1995, Clavo et al. observed an increase in [^18^F]-FDG uptake in two human malignant cell lines exposed to low oxygen concentrations and concluded that this radiotracer could partly reflect tumor hypoxia (20). Zhao et al in a study of [^18^F]-FDG biodistribution in rat inoculated hepatoma cells showed that the uptake pattern of [^18^F]-FDG, as well as the expression of glucose transport proteins and hexokinase are related to the presence of HIF-1α, using autoradiography and immunostaining (21). In 2008, Dierckx and Van De Wiele suggested that there was a correlation between glucose metabolism identified by [^18^F]-FDG and the level of oxygen in cells (22).

However, several studies evaluating the correlation between [^18^F]-FDG uptake and the level of hypoxia obtained contradictory results (23, 24). In 2006, Zimny et al. showed that hypoxia in head and neck tumors affects glucose metabolism but [^18^F]-FDG PET cannot reliably differentiate hypoxic from normoxic tumors (25). Gagel et al. showed that there was no correlation between [^18^F]-FDG PET and hypoxia assessed by [^18^F]-FMISO in patients with non-small cell lung cancer (26) and Kronke et al. made the same findings in HNC (27). In 2007, Gagel et al. showed the lack of correlation of direct comparison of [^18^F]-FDG uptake and hypoxia determination using a polarographic O_2_ sensor in 38 patients with head and neck cancer (28). Lopci et al. hypothesize that these results are understandable because under conditions of low oxygen concentrations, cells switch their metabolic pathway of ATP production to anaerobic glycolysis, also known as the Pasteur effect (24). In hypoxic tumor cells, there is an overlap between [^18^F]-FDG uptake due to aerobic glycolysis, known as the Warburg effect (29), and anaerobic glycolysis (30, 31). Also, the HIF-1α protein that can be observed in non-hypoxic tumor regions suggests that other factors may indirectly influence glucose metabolism and [^18^F]-FDG uptake in these regions (32). In a recent 2021 study, Thureau et al. compared the uptake rates between [^18^F]-FDG, [^18^F]-FMISO, and [^18^F]-FAZA in lung cancer. Nineteen patients were included in this study and acquisitions of the two hypoxic tracers were performed over a median period of 2.1 days in random order. The results showed the lack of correlation between [^18^F]-FDG PET and PET with the two hypoxia radiotracers confirming that [^18^F]-FDG provides different information and is independent but complementary to the hypoxia radiotracers (33).

If it has been clearly shown the hyperfixation of [^18^F]-FDG in solid tumors (34), these up- or down-regulation of [^18^F]-FDG due to antagonistic phenomena shows that this hyperfixation is not specific to tumor hypoxia. As [^18^F]-FDG PET is a standard diagnostic test in solid tumor cancers, as well as in therapeutic follow-up, it is readily available and could be used in combination with other hypoxia tracers to obtain a comprehensive assessment of tumor characteristics.

#### 3.3.2. Redox potential

##### 3.3.2.1. Nitroimidazole compounds

These compounds undergo reduction in oxygen-depleted tissues, producing metabolites that can be uptaken within cells. The nitroreductases involved include xanthine oxidases, lipooxygenases and NADPH oxidases, which release a metabolite that has a single electron. This radical will react with intracellular proteins to form a covalent bond. It is this mechanism that is used in the use of radionuclide labeled nitroimidazole tracers that allow direct measurement of tissue oxygenation levels. Numerous tracers have been used, the leading one being [^18^F]-FMISO.

##### 3.3.2.2. Others (Cu-ATSM)

Copper transport proteins do not appear to be involved in the cellular uptake mechanism of Cu(II)-ATSM and remains unclear. An intriguing question is whether the uptake of ATSM-Cu(II) depends upon the cell types or the cellular oxygen levels. Once ATSM-Cu(II) enters the cell, the rate of copper dissociation from ATSM ligands depends largely on cellular oxygen levels. In normoxic cells, Cu(II)-ATSM, which has a low redox potential, is not reduced to Cu(I) or the Cu(I)-ATSM intermediate; therefore, it is not retained intracellularly, remains solvable in aqueous media, and is easily cleared from cells. In mirror, hypoxic cells may cause the reduction of ATSM-Cu(II) due to the aggressive reducing conditions. Copper accumulation in cells depends on the reduction of copper(II) to copper(I) in cytosol but also on the extraction rate of ATSM-Cu(II) from circulating blood. In hypoxic cells, copper(I) will tend to decomplex from ATSM and precipitate as copper sulfide.

#### 3.3.3. Intracellular pH variations

Hypoxia leads the cell to use anaerobic metabolism to function. This metabolism produces less energy than aerobic metabolism (2 ATP vs. 36 per glucose consumed) and leads to the formation of lactic acid causing acidification of intracellular pH. This pH change is detrimental to the long-term survival of the cell.

Carbonic anhydrases are transmembrane zinc metalloenzymes that allow the formation of bicarbonate from carbon dioxide by hydration. Carbonic anhydrase IX (CA IX) is the only isoenzyme of this family associated with tumor proliferation. It regulates the pH of cells in a state of hypoxia since its coding gene (CA9) is located downstream of the HRE sequence. In particular, its expression has been found in 94% of clear cell renal cell carcinomas. Thus, a specific antibody in this indication has been developed for treatment (girentuximab, Rencarex^®^) but without clinical benefits (35). A labeled derivative has been developed for diagnosis ([124I]-girentuximab, Redectane, CA9-scan).

The detection of CAIX expression in PET imaging is potentially interesting for the assessment of tumor hypoxia because of its relatively high expression on the cell surface and its prolonged presence in hypoxic tissues, in contrast to HIF-1α protein. Correlations have been reported in the literature between tracer uptake and CAIX expression, but the data provided are still too limited and based only on preclinical methodologies to confirm their value in the clinical identification of tumor hypoxia.

#### 3.3.4. Water diffusion in tissue

Dunn et al. have shown that the apparent diffusion coefficient (ADC) of water in chronically hypoxic tumor tissue is directly related to tumor pO_2_. The relationship between tumor oxygenation and the ADC of water would be valuable for discriminating the oxygenation status of viable, hypoxic, and necrotic tissue and for monitoring treatment. MRI mapping may be used as well as [^15^O]-H_2_O diffusion to indirectly find the regions of hypoxia in tumors.

**FIGURE 1:**
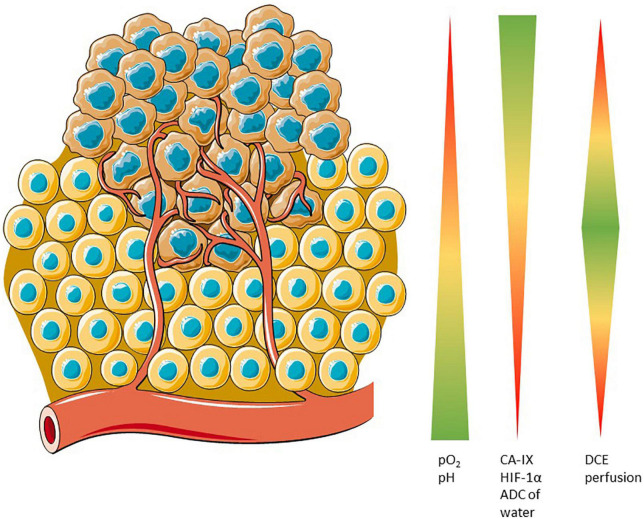
Potential cellular targets for hypoxia imaging. pO_2_ and pH are closely related because of the lack of oxygen leads to an anaerobic metabolism in cell which acidifies the cytoplasm. In hypoxic conditions, CA-IX is overexpressed because of HIF-1α deregulation. Apparent Diffusion Coefficient is closely related to the pO_2_ and Dynamic Contrast Enhanced is sensitive to fine changes in the vasculature.

The main targets of hypoxia imaging are summarized in the Figure 1.

## 4. Medical imaging

**FIGURE 2:**
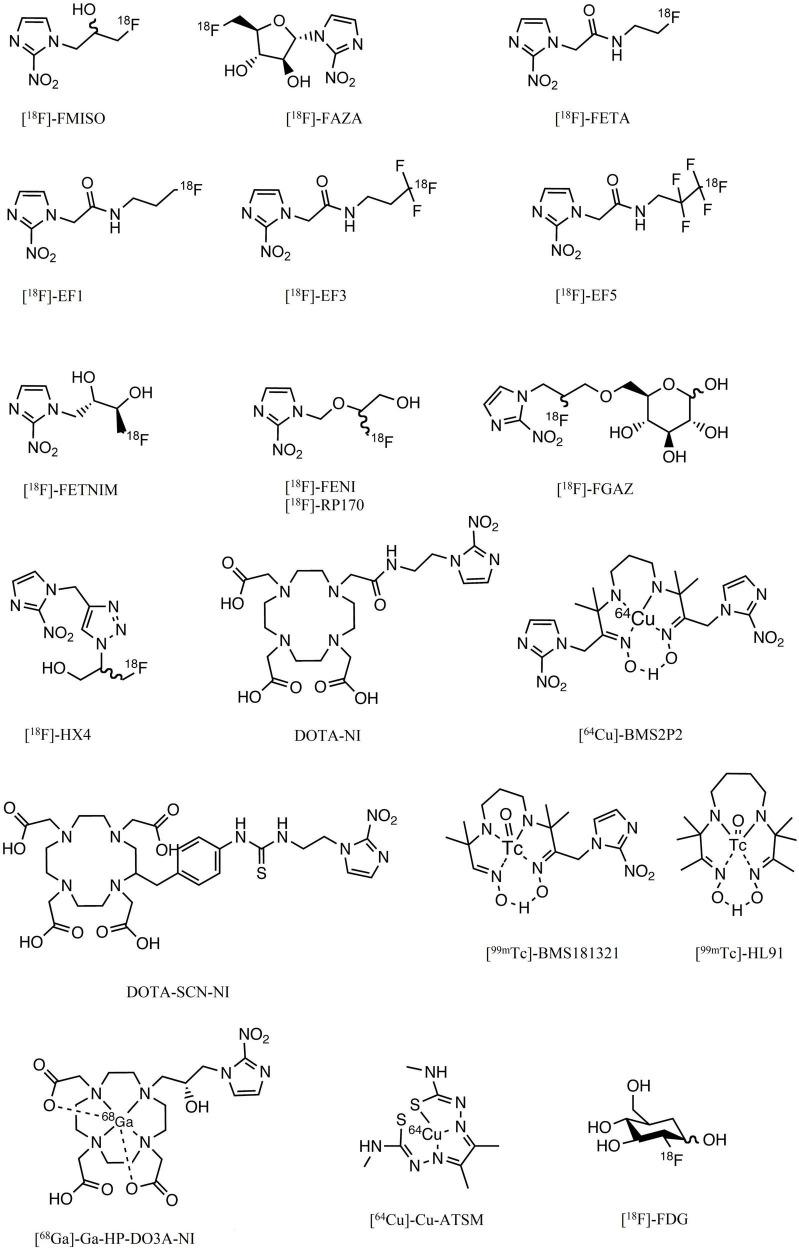
Chemical structures of the main compounds used or having been used in the assessment of hypoxia.

Functional PET imaging can provide insight into the complex interactions between tumor and host during antineoplastic therapy. Specific tracers can provide different biological information. For instance, FDG can measure glucose metabolism and indirectly inflammation, FMISO is able to detect hypoxic tumor subvolumes, Anti-CAIX can evaluate the variation of intracellular pH and now some complex quantitative parameters such as dynamic PET, Ki, or Vd could improve the knowledge of the tumor microenvironment. Other imaging techniques can provide additional information. For example, CT improves the accuracy of localization and provides information on tissue density. But above all, MRI can provide information on the diffusion of water in the tissues, the quantity of free oxygen or oxyhemoglobin in addition to anatomical information. The main chemical structures are presented in the Figure 2.

### 4.1. [^18^F]-FDG PET/CT

[^18^F]-FDG is a tracer of the first steps of glucose metabolism, therefore, an enhanced uptake is not limited to tumor tissues. Physiological uptakes can be observed in skeletal muscle after exercise or contraction but not at rest, in central nervous system, into myocardium, in bladder and ureters due to urinary excretion, in liver, sometimes in stomach, barely in lungs.

**FIGURE 3:**
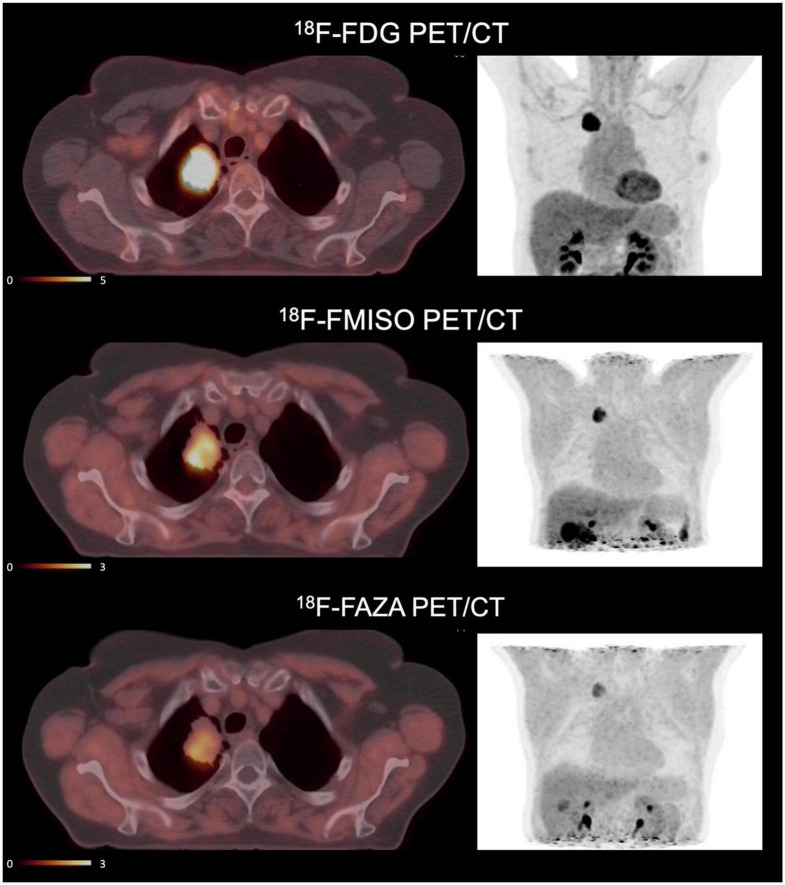
Example of PET imaging of a patient with [^18^F]-FDG, [^18^F]-FMISO and [^18^F]-FAZA. This patient with non-small cell lung cancer is included in a clinical trial “RTEP6” and signed an informed consent. Hypermetabolic regions are visualized by [^18^F]-FDG and hypoxic regions by [^18^F]-FMISO and [^18^F]-FAZA. Standardized uptake values (SUV) of [^18^F]-FDG are of an order of magnitude 5 times higher than SUV of [^18^F]-FMISO and [^18^F]-FAZA. The accumulation of tracers does not necessarily occur in the same tissue territories. The uptake of [^18^F]-FAZA is lower than [^18^F]-FMISO (33).

The [^18^F]-FDG uptake seems not associated to the presence of tumor hypoxia via the upregulation of glucose transporter 1 by hypoxia-inducible factor 1 in patients. [^18^F]-FDG and a hypoxic tracer such as [^18^F]-FMISO or [^18^F]-FAZA provide different and perhaps complementary information to delineate targets volumes for radiotherapy. Figure 3 illustrates this complementary information in a patient with NSCLC before treatment. The dose commonly injected is 1.5–4 MBq/kg and PET scanning usually starts from 60 min after injection at rest.

### 4.2. Redox potential imaging

#### 4.2.1. Nitroimidazole compounds

##### 4.2.1.1. [^18^F]-FMISO

[^18^F]-FMISO is the reference and most studied tracer. Mostly preclinical studies began between 1999 and 2005. Chapman et al. were the first to propose nitroimidazoles as biomarkers of hypoxia (36, 37). The first studies were performed after tritium and fluorine labeling. These studies showed *in vitro* (V-79, EMT-6, RIF-1, canine osteosarcoma lines) and *in vivo* on mouse tumors (AH019A) the good uptake of the tracer in hypoxic cells (38). The authors also showed a correlation between [^18^F]-FMISO binding and the proportion of hypoxic cells (39). Mathematical models or the Standard Uptake Value (SUV) study have been used to quantify the hypoxic fraction in animal and human imaging (40). All methods showed an underestimation of hypoxia in severe hypoxic conditions (pO_2_ 2–3 mmHg), probably explained by an impairment of biochemical functions. Several studies have compared the biodistribution of [^18^F]-FMISO with the use of pimonidazole and carbonic anhydrase IX (CA IX) expression in immunohistochemistry in rhadmyosarcoma models. Studies have shown comparable binding between [^18^F]-FMISO and pimonidazole. The correlation of uptake of [^18^F]-FMISO and hypoxia-related gene expression in HNC seems weak (41). Studies that compared the performance of [^18^F]-FMISO versus fluoro-2-deoxy-D-glucose ([^18^F]-FDG), using Eppendorf electrodes as a reference, showed superiority for [^18^F]-FMISO in demonstrating hypoxia (25, 26, 28, 42).

Physiologically, [^18^F]-FMISO go through the blood-brain-barrier and accumulates in the normal brain tissue, and due to this lipophilic nature it can be excreted by the hepatobiliary system. The clearance from the blood is slow.

In practice, [^18^F]-FMISO PET imaging is performed after injection of 120–450 MBq (2–5 MBq/kg) of [^18^F]-FMISO. A relatively long latency of 2–4 h post-injection due to the slow clearance from the blood and relatively long acquisitions (5–8 min) centered on the lesion area are required. It is not necessary to be fasting (43). The usual hypoxic threshold is 1.6 tumor-to-muscle ratio (TMR) or 1.2–1.4 tumor-to-blood ratio (TBR).

Koh et al. pioneered the use of [^18^F]-FMISO in humans (44), and it was subsequently used in gliomas (45–49), HNC (13, 25, 42, 50, 51), oral squamous cell carcinoma (52), bronchopulmonary tumors (33, 40, 53–55), renal tumors (56), and in sarcomas (57). The dynamic [^18^F]-FMISO-PET is predictive of outcome in patients with HNC treated by radiotherapy and paved the way of a dose-painting strategy (58, 59). In contrast, this accumulation was variable in sarcomas (13, 60) and absent in pancreatic cancer (61). Furthermore, a large and non-specific accumulation in normoxic tissues compromises the study of hypoxia in rectal cancer (62).

Dynamic imaging were performed by Schwartz et al. in 16 patients with NSCLC scheduled for definitive radiation therapy in order to definitively differentiate the uptake and perfusion of [^18^F]-FMISO because of the slow clearance of this tracer in normal tissues (63). However, this study showed there is not always concordance between the classic parametric descriptors of hypoxia such as the tumor-to-blood ratio and the rate at which [^18^F]-FMISO is trapped in tissues as used to be a surrogate hypoxia metric. These inconsistencies might be a consequence of underlying different tumor pathologies such as chronic hypoxia and ribbon-like hypoxia that is observed by immunohistochemical staining but not resolved by PET imaging.

Vera et al. performed a pilot study (RTEP4) with three different tracers (metabolism, [^18^F]-FDG; hypoxia, [^18^F]-FMISO and proliferation [^18^F]-FLT) in five patients with NSCLC treated by radiation therapy. The acquisitions were performed before and during treatment and showed correlations between the three tracers (54). This research group performed a multicentric study with the aim to increase selectively the radiation therapy dose in hypoxic tumor areas. Fifty-four patients were included, 34 were [^18^F]-FMISO positive and 24 of whom received escalated doses in hypoxic areas without exceeding the tolerance in organs at risk. After 3-year follow-up, the boost radiotherapy seemed to improve the overall survival by 11 months in patients with hypoxic tumor, but due to the small sample size this result should be confirmed on a larger population (64–66). Another criticism would be the definition of the threshold used to define hypoxic volume because a fixed threshold of SUV > 1.4 was retained in the study. Vera et al. also compared the uptakes of [^18^F]-FDG, [^18^F]-FMISO and [^18^F]-FAZA before surgery in lung cancer. [^18^F]-FMISO and [^18^F]-FAZA were strongly correlated and [^18^F]-FMISO images were better than [^18^F]-FAZA in the same patient’ conditions (33).

Zschaeck et al. have reported in 2021 the first clinical evidence that tumor hypoxia is inversely correlated with radiation induced inflammation in head and neck cancer using [^18^F]-FDG and [^18^F]-FMISO (67). Zschaeck et al. have performed a meta-analysis of [^18^F]-FAZA and [^18^F]-FMISO hypoxia PET scans in head and neck cancer patients. They found that the prognostic value of hypoxia PET was robust regardless of the hypoxia tracer, the imaging site, and patient status but a hypoxia specific treatment seems not improve outcome in *post-hoc* analyses (68). Löck et al. found that the residual tumor hypoxia during radiochemotherapy assessed by [^18^F]-FMISO PET denotes treatment resistance in HNC and is a biomarker for selection of patients at high risk of loco-regional recurrence after treatment (69).

##### 4.2.1.2. [^18^F]-FAZA

[^18^F]-fluoroazomycin-arabinofuranoside ([^18^F]-FAZA) is more hydrophilic than [^18^F]-FMISO (70). As a result, clearance kinetics are faster, which would result in an improved ratio of hypoxic tumor to reference tissue (71). Biodistribution and dosimetry have been studied in 5 NSCLC patients, and indicated a favorable radiation risk profile (72). Developed in 2002, several clinical studies have since successfully evaluated [^18^F]-FAZA uptake to visualize tumor hypoxia in gliomas (73), brain lymphomas (73), lung tumors (72, 74, 75), head and neck tumors (68, 76–78), cervical cancer (79), and rectal tumors (80). [^18^F]-FAZA did not detect hypoxia in kidney cancer nor in distant metastases (81). It has been shown in these studies that the results are comparable with those obtained with [^18^F]-FMISO (highly correlated hypoxic areas), with often better early contrast in favor of [^18^F]-FAZA between hypoxic and normoxic tissue. In general, [^18^F]-FAZA seems to have more potential applications than [^18^F]-FMISO (14). However, a study by Thureau et al. showed that the images obtained with [^18^F]-FMISO and [^18^F]-FAZA in lung cancer were highly correlated and that [^18^F]-FMISO images were more contrasted than [^18^F]-FAZA images between hypoxic and normoxic tissues in the same patient (33). [^18^F]-FAZA and [62Cu]-ATSM have been compared in NSCLC. Hypoxic PET images were acquired after 2 h the injection of [^18^F]-FAZA (45 patients) and 10–20 min after injection of [62Cu]-ATSM (22 patients). Uptakes of hypoxic tracers have been correlated (82). The injected dose is usually 4 MBq/kg or a fixed activity of 370 or 400 MBq. PET scanning usually starts from 120 or 240 min after injection. Physiologically, FAZA does not cross the blood-brain barrier because of hydrophilicity and has a relatively fast urinary excretion. The reader can find more information in the Savi et al.’s study (72).

##### 4.2.1.3. [^18^F]-FETNIM

[^18^F]-Fluoroerythronitromidazole ([^18^F]-FETNIM) also has better hydrophilicity than [^18^F]-FMISO allowing rapid renal clearance and low hepatic uptake (83, 84). Compared to [^15^O]-H2O, the initial uptake of [^18^F]-FETNIM was governed by blood flow and retention in hypoxic tissue occurred after 60–90 min after injection (85). This tracer is therefore eliminated more rapidly from normoxic tissues which theoretically allows a higher tumor to normoxic tissue ratio. Clinical studies in patients with head and neck (86–88), esophageal (89), lung (90), glioma (91), and cervical cancer (92) have shown that [^18^F]-FETNIM PET is feasible and useful for hypoxia imaging. However, a superior benefit over [^18^F]-FMISO was not shown because the tumor-to-blood ratio of this tracer was not significantly higher (93, 94). The added value of [^18^F]-FETNIM over [^18^F]-FMISO is currently still debated. The injected dose is usually 3.7 MBq/kg; the acquisition begins from 120 min after injection, in the later phase the images tended to fade (i.e., 180 min and more after injection).

##### 4.2.1.4. [^18^F]-RP-170

[^18^F]-RP-170, another hypoxic 2-nitroimidazole radiosensitizer, has also been labeled with fluorine 18 (95). It has shown, in patients with gliomas, significant correlations between SUV and oxygen partial pressure measurements (96). Studies of brain and lung tumors indicated a higher SUV for hypoxic tissue than for normoxic tissue, with a ratio of 1.7 measured 1 h post-injection (97, 98). The shorter time interval between injection and acquisition, as well as better contrast of hypoxic tissue compared with [^18^F]-FMISO, suggests that [^18^F]-RP-170 could potentially have clinical applications. The injected dose is usually a fixed activity of 185 or 370 MBq. The PET scanning starts from 120 min after injection.

##### 4.2.1.5. [^18^F]-EF3

Several molecules from EF family were synthesized, EF1, EF3 and EF5. EF1, which seemed promising, was quickly abandoned because of its rapid defluorination. [^18^F]-EF3 was particularly studied by Gregoire et al. and evaluated in ENT tumors. [^18^F]-EF3 showed faster clearance than [^18^F]-FMISO with images taken 4 h postinjection and uniform distribution in healthy tissue comparable to [^18^F]-FMISO (99, 100). The injected dose is usually a fixed activity of 370 MBq (and up to 1100 MBq in the clinical trial of Mahy et al.). This compound is mainly eliminated in the urine via the kidneys, and a lower extent in the intestines via the liver has been observed.

##### 4.2.1.6. [^18^F]-EF5

Another analogue of nitroimidazole compounds has been proposed in the literature. It is 2-(2-nitro-1H-imidazol-1-yl)-N-(2,2,3,3,3-penta-fluoropropyl)-acetamide labeled with fluorine 18 ([^18^F]-EF5). More lipophilic than previously described compounds, [^18^F]-EF5 has a higher octanol-water partition coefficient, which allows for easier passage through the cell membrane by passive diffusion. This could also improve the uniformity of tumor uptake and tracer distribution (101). [^18^F]-EF5 has shown the ability to detect tumor hypoxia in head and neck cancer (102), cervical and brain tumors (101, 103, 104). However, its labeling chemistry appears to be difficult and the potential advantage over [^18^F]-FMISO seems negligible (14). [^18^F]-EF5 was studied in NSCLC and the tracer uptake was correlated to tumor hypoxia (105, 106). This tracer was studied in ovarian cancers but this indication appeared to be limited due to the relatively weak uptake in tumors and the biliary excretion may impose limitations on assessing hypoxic tumors located near to the intestine (107). The injected dose is usually 4 MBq/kg or a fixed activity of 300 MBq. Its excretion is predominantly urinary via the kidney with a lower extent in bowel via the liver. It has been observed a uniform distribution of [^18^F]-EF5 in normal brain due to its lipophilicity (101).

##### 4.2.1.7. [^18^F]-HX4

In 2011, Dubois et al. synthesized ^18^F-Flortanidazole (^18^F-HX4). It contains a 1, 2, 3-triazole moiety introduced by a “click chemistry” (108). This process allows for rapid and efficient product synthesis by assembling small units together through heteroatom linkages. The 1,2,3-triazole moiety makes ^18^F-HX4 more hydrophilic, which increases its renal clearance. These features therefore contribute to a more rapid decrease in signal from normoxic tissues. A relationship has been found between pO_2_ and [^18^F]-HX4 uptake in NSCLC (109). The absence of toxicity has been demonstrated (110) and kinetics can be described by a reversible two-tissue compartment model (111). A simulation study has shown that, compared to [^18^F]-FMISO and [^18^F]-FAZA, [^18^F]-HX4 has a higher tumor-to-blood ratio but the largest patient-to-patient variation (112). In mice, [^18^F]-FAZA or [^18^F]-HX4 are more sensitive to acute hypoxia whereas [^18^F]-FMISO uptake is influenced by reoxygenation (113). In head and neck cancers, [^18^F]-HX4 yielded similar tumor baseline tissue values to ^18^F-FMISO at relatively early post-injection time points, indicating a potential advantage of shorter acquisition time (114). However, a study in lung cancer patients suggested that PET imaging performed at 4 h post-injection would provide superior contrast compared with 2 h post-injection (115). This tracer can be used as a hypoxia PET tracer in NSCLC and HNSCC, and may be used to monitor the modification of hypoxic areas during radiotherapy or treatment intensification (115–119). Zegers et al. performed two ^18^F-HX4 PET scans during the same week and before treatment in patients with lung and head and neck cancer showing that ^18^F-HX4 PET provides reproducible and stable results over time (120). The injected dose is usually a fixed activity of 400 MBq and PET/CT imaging is performed at 120 or 240 min post-injection. The 240 min. p.i. time point is related to a plateau phase in tracer uptake. The hypoxic region was based on a threshold of 1.4 ± 0.2 tumor-to-blood ratio (115).

##### 4.2.1.8. [^18^F]-FGAZ

This compound is a combination of azomycin and 2-nitroimidazole. Unfortunately, it is not a suitable tracer of hypoxia *in vivo* (121).

##### 4.2.1.9. [^68^Ga]-Nitroimidazole

[^68^Ga] is a radionuclide produced by a Germanium-68/Gallium-68 generator available in the radiopharmaceutical laboratory of nuclear medicine services. It has excellent coordination chemistry with several chelating agents, allowing rapid and economical radiolabeling. The main strong chelators of gallium are cyclic like NOTA, DOTA, DO3A DOTAGA or NODAGA (122), but some are acyclic like dedpa and derivatives (123). Based on the bioreductive properties of nitroimidazole, [^68^Ga]-labeled nitroimidazole derivatives have been synthesized and preclinically studied as promising candidates for hypoxia PET imaging (124–127). The pre-clinical studies with xenografted tumors have shown that [^68^Ga]-labeled hypoxia tracers give comparable results to fluorinated hypoxia tracers with greater ease of production. These results encourage further clinical research into the imaging and quantification of hypoxic diseases with [^68^Ga]-labeled nitroimidazole PET/CT (128). A clinical study has been conducted by Bresser et al. in patients to explore hypoxia in tuberculosis. This disease is compared to a bacterial tumor. In this case, intravenous administration of 74–185 MBq [^68^Ga]-nitroimidazole was performed and imaging occurred 60–90 min post-injection. The primary excretion route was the urinary tract, and a lower extent in liver and intestines was observed. In hypoxic regions a low-grade uptake of this tracer was observed (SUV_mean_ of 0.47) but the median lesion-to-muscle ratio was 1.7 (129).

##### 4.2.1.10. [^64^Cu]-BMS2P2

This compound is similar to [^64^Cu]-BMS181321. This is a bisnitroimidazole probe and designed to have an enhanced uptake in hypoxic cells. However, it seems that the high lipophilicity resulted in significant distribution in the intestine and liver and impeded clearance in normal tissue (130).

#### 4.2.2. Other compounds

##### 4.2.2.1. [*Cu]-ATSM

Cu(II)-ATSM diffuses into the cell due to its high membrane permeability and low redox potential. In the cell, the rate of copper dissociation from ATSM ligands depends largely on cellular oxygen levels. In normoxic cells, Cu(II)-ATSM, which has a low redox potential, is not reduced to Cu(I) or the Cu(I)-ATSM intermediate. Therefore, it is not retained intracellularly. It remains soluble in aqueous media and is easily cleared from cells. On the other hand, hypoxic cells can cause the reduction of Cu(II)-ATSM due to reducing conditions, so the radioactive copper remains in the cell. Its cellular accumulation depends on the reduction of copper(II) to copper(I) in the cytosol, but also on the rate of extraction of ATSM-Cu(II) from the circulating blood. In hypoxic cells, copper(I) will tend to decomplex from ATSM and precipitate as copper sulfide. Compared to [^18^F]-FDG and nitroimidazole fluorinated tracers, Cu-ATSM appears to target hypoxic areas of the tumor more specifically in PET imaging (131–135).

In the late 1990s, Fujibayashi et al. successfully studied the accumulation of [62Cu]-ATSM under hypoxic cardiac perfusion conditions in rats (132). In humans, tumor-specific retention of Cu-ATSM has been shown for head and neck (136, 137), lung (138–140), rectal (141), cervical (142), and glioma cancers (143) with a good concentration ratio between hypoxic tumor and healthy tissue. However, the exact mechanism of Cu-ATSM selectivity toward hypoxia remains unclear (131, 135, 144). An intriguing question is to know whether the mechanism of the uptake of Cu(II)-ATSM depends on cell types, cellular oxygen levels, reductases in blood or dissociation of Cu-ATSM into ionic copper salts in blood.

Nevertheless, Cu-ATSM has several advantages over other tracers for tumor hypoxia imaging, including simpler synthesis and radiolabeling methodology and faster clearance of normal tissue, allowing for shorter time frames and image acquisition with potentially higher contrast between hypoxic and normoxic tissue. Studies have shown other advantages based on ATSM-labeled copper isotopes when used for hypoxia imaging as a monitor of therapeutic response (136, 145) and predictive of outcome in patients with HNC (146). [^64^Cu]-ATSM can also be used for internal radiation therapy (133, 134, 147, 148).

The injected dose for [^64^Cu]-ATSM is usually a fixed activity of 300 (50–400) MBq and PET/CT imaging is performed at 16 or 18 h. post-injection (149, 150). For [^61^Cu]-ATSM, a fixed activity of 111 MBq (3 mCi) can be injected 2–3 h before imaging (151).

##### 4.2.2.2. Amineoxime

[^99m^Tc]-BMS181321 is a chemical compound in which a propylene amineoxime was associated to a 2-nitroimidazole. This compound can also be labeled with [^64^Cu]-copper. The lipophilicity of this compound is high and hindered the clearance from normoxic tissue. Another compound from the series of amineoxime chelators was highlighted, the butyleneamineoxime (BnAO, or HL91). It was initially used as a control compound for BMS181321 but it showed high and specific accumulation in hypoxic cells *in vitro* with a good hydrophilicity permitting a rapid clearance from blood in animals. However, inverse correlation between [^99m^Tc]-HL91 uptake and pO_2_ remains controversial in clinical use. [^99m^Tc]-HL91, like [^99m^Tc]-HMPAO, might rather be considered as a perfusion tracer.

### 4.3. Intracellular pH variations

#### 4.3.1. [^89^Zr]-cG250/[^124^I]-cG250

Oosterwijk et al. developed the G250 antibody (girentuximab) in 1986 for identification of CAIX (152). In 2007, Divgi et al. showed that the 124 iodine-labeled antibody ([^124^I]-cG250) could accurately identify clear cell renal cell carcinoma and could therefore be used as a tracer in PET imaging (153), but CAIX expression was not always related to hypoxia (154). In 2010 in a preclinical study of head and neck carcinomas, Hoeben et al. showed a spatial correlation between [^89^Zr]-cG250-F(ab’)2 binding and CAIX expression at the microscopic level, suggesting sufficient tumor uptake of the tracer with accurate microscopic localization of hypoxia (155). The injected dose for [^89^Zr]-girentuximab is usually a fixed activity of 37 MBq and PET/CT imaging is performed at 24, 72 and up to 168 h. post-injection. The best tumor-to-background ratio is observed at late acquisition in hypoxic tumors expressing CAIX. The biodistribution is similar to that of other [^89^Zr]-mAb with a slow clearance from liver, heart, kidney and blood pool. The uptake from renal cell carninoma (assumed to be hypoxic) is rather slow in the clinical trial performed by Merkx et al. (156).

#### 4.3.2. Sulfonamide-based CA-IX imaging agents

These compounds can be related to acetazolamide, a pan-carbonic anhydrase inhibitor and pharmacomodulated to reach a specificity against CA-IX. Recent studies have explored these compounds in PET by labeling them with Gallium 68 ([^68^Ga]-US2) or fluorine 18 without directly correlating them to tumor hypoxia (157, 158). Very recently, Nakashima et al. also confirmed that [^68^Ga]-labeled imidazothiadazole sulfonamide derivatives can assist in the visualization of hypoxic tumor tissue 2 h after intravenous injection (159).

### 4.4. Magnetic resonance imaging

Dunn et al. have shown that the ADC of water in tumor tissue with chronic hypoxia is directly related to tumor pO_2_ (160). This can be visualized in MRI by 3D mapping of the apparent diffusion coefficient (ADC) of water in the tissue. The relationship between tumor oxygenation and free water ADC would be valuable in distinguishing the oxygenation status of viable, hypoxic and necrotic tissue and in assessing the efficacy of treatment.

Diffusion MRI reflects the degree of mobility of water molecules diffusing into tissues highlighting tissue organization at the microscopic level. While pure water exhibits random isotropic molecular diffusion, the movement of water molecules *in vivo* is limited by cell membranes and other obstacles. In 1986, the first study in humans showed that a measure of molecular diffusion has lower values for solid tumors than for healthy tissues, because molecular movement is restricted due to greater cellularity (161). In this study, Le Bihan et al. introduced one of the most essential parameters of this technique, the apparent diffusion coefficient (ADC). The ADC is the diffusion coefficient reflecting the tissue microstructure surrounding the diffusing water molecules (restriction in closed spaces, tortuosity around obstacles, hindrance, etc.), as opposed to the free and unrestricted diffusion of water in free environments.

The potential link between ADC measurement and hypoxia has been investigated in preclinical studies, where temporal changes in ADC measurements in hypoxic brain lesions in rats have been found (162, 163). To the best of our knowledge, no preclinical or clinical studies have been able to establish a direct link between ADC measurement and the level of tumor hypoxia. ADC has been described as a tool to define the level of tumor hypoxia when combined with other relaxometric (T2 mapping) or perfusion MRI sequences (164–166).

The diffusion of [^15^O]-H_2_O estimated by PET imaging to indirectly find regions of hypoxia in tumors has also been proposed in the literature, but this technique is challenging due to the very short half-life (2 min) of 15-oxygen and perfusion is not always correlated with hypoxia (46, 167).

The partial pressure of oxygen has an influence on the T1 and T2 relaxation times in MRI. The physicochemical environment has an influence on the T1 and T2 relaxation times of tissues in nuclear magnetic resonance. In particular, oxyhemoglobin contained in oxygenated red blood cells is a diamagnetic molecule, whereas deoxyhemoglobin contained in deoxygenated red blood cells is paramagnetic (168). An increase in the concentration of deoxyhemoglobin leads to an acceleration of the spin-lattice relaxation rates and thus to a decrease in the T1 and T2 relaxation times. This phenomenon can be assessed by MRI techniques, either by using T2*-weighted sequences to demonstrate the BOLD (Blood Oxygen Level Dependent) effect (169), or by obtaining T1 and T2 maps (164, 170). As for the diffusion technique, the relation between T1 and T2 values would be useful to discriminate normoxic, hypoxic and necrotic tissues.

MRI acquisition techniques to visualize the BOLD (Blood Oxygen Level Dependent) effect were developed in the early 1990s by Ogawa et al. by exploiting differences in nuclear magnetic resonance (NMR) transverse relaxation times without correction for inhomogeneities in the main MRI field, T2* (169). This application has been developed for brain imaging in combination with neuronal activation induced by a so-called functional MRI stimulation, designed to dynamically modify the ratio between oxygenated and deoxygenated hemoglobin.

Sensitive changes in the signal thus allow mapping of tissue oxygenation variations (171). In a preclinical study in 2011, Christen et al. showed a correlation between oxygen saturation levels and blood gas analysis (172). These results were confirmed in humans in 2012 (173). Recently, a preclinical study with a xenograft model of colon cancer in mice showed that BOLD was positively correlated with HIF-1α, indicating that T2*-weighted sequences might be predictive of tumor hypoxia (174). BOLD imaging has mapped chronic hypoxic regions in prostate carcinoma with some success (175), but signal changes do not correlate well with absolute pO2 level. Indeed, the BOLD image is sensitive to multiple factors, such as blood volume which depends on vessel caliber, motion artifacts, magnetic susceptibility, magnetic field inhomogeneities or the acquisition protocol adopted which may cause ambiguities in interpretation (16, 176). Despite encouraging data, there is currently no evidence to suggest that the BOLD effect can be used in tumor hypoxia.

In 1971, Damadian demonstrated that the T1 and T2 relaxation times of liver tumor tissue in mice are significantly increased compared to those of healthy tissue (177). This work demonstrated for the first time that NMR techniques could distinguish healthy tissue from tumor tissue by measuring T1 and/or T2.

MRI is usually based on T1-, T2-, or ρ-weighted (proton density) sequences. In order not to have a composite contrast that is difficult to interpret, it is preferred to have a contrast that depends mainly on a single intrinsic tissue parameter. Nevertheless, it is possible to perform quantitative magnetic resonance imaging (qMRI) allowing a true measurement of intrinsic parameters. qIRM is based on measurement techniques inspired by NMR relaxometry methods. Initially little used because of the prohibitive duration of acquisition times, the improvement of the technical performances of MRI imagers in recent years have allowed the development of new acquisition methods and to reduce the acquisition times of quantitative imaging. It has been shown that this approach allows the characterization of tissues and pathologies (178). In particular, in oncology, it has been shown in prostate cancer, that the analysis of T2 relaxation time would allow to differentiate normal and pathological tissues (179, 180). Recently, a pre-clinical study in mice showed that T2 mapping associated with ADC were correlated in the characterization of tumor hypoxia (164).

Despite these encouraging results, to our knowledge, no clinical study has explored the potential value of using qIRM to demonstrate and quantify tumor hypoxia. However, we believe that the results obtained in recent years by these measurement methods are promising.

#### 4.4.1. Perfusion imaging

To compensate for the lack of O_2_ within the tumor, pro-angiogenic factors are activated in the tumor cells creating new blood vessels from pre-existing ones (181). However, these new vessels are abnormal and have abnormal branching, irregular diameter and fragile endothelial wall leading to impaired blood perfusion compared to healthy tissue (182). Oxygen supply is primarily governed by blood perfusion, whereas oxygen consumption is primarily determined by the respiratory function of the tissue and, therefore, cell density (12). Therefore, tumor hypoxia is expected to be found in areas of low blood perfusion and/or high cell density.

Perfusion imaging studies the microcirculation within blood capillaries. It provides morphological and functional data. As in CT, the principle of this imaging is to analyze in 3D the kinetics of the passage through the capillary walls of a gadolinium chelate after its injection into the vascular system. Perfusion is then studied within the tumor, highlighting perfused tumor areas, or poorly visible areas showing perfusion defects within the tumor. Tofts et al. proposed the Dynamic Contrast Enhanced (DCE) perfusion method in MRI using quantitative compartmental analysis, sensitive to fine changes in the vasculature by analyzing permeability and perfusion parameters as well as vascular and extracellular volume fractions. Therefore, DCE-MRI can potentially be used to identify hypoxic regions (183).

**FIGURE 4:**
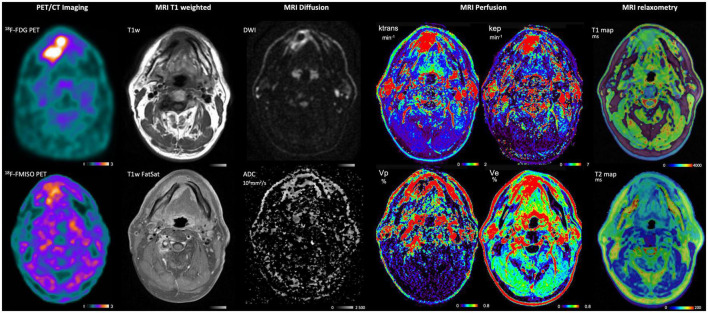
PET and MRI imaging of a patient with HNC included in the study “RTEP8”. Multimodality imaging has been performed with [^18^F]-FDG (metabolism), [^18^F]-FMISO (hypoxia) for the PET imaging. MRI T1 weighted, MRI diffusion, MRI Perfusion and MRI relaxometry have been performed at the same time. This patient had a lesion of the right lower paramedian gum. The T1-weighted sequence showed a hyposignal infiltration and a hypersignal of the lesion with fat saturation. The lesion was in diffusion hypersignal with restriction of free water diffusion. Perfusion sequence Vp showed a contrast within the lesion proving a lack of vascularization in the tumor and might be correlated with the [^18^F]-FMISO uptake. T1 and T2 relaxation time velocities are decreased within the lesion.

Several studies have correlated DCE-MRI measurements with HIF-1α, oxygen level, and immunohistochemical analysis of the hypoxic fraction (184–188). However, correlations are generally weak. Hammond et al. explain this by the fact that the studies measure significantly different MRI parameters, all of which have a partial and variable relationship with perfusion (189). Also, chronic hypoxia, as well as transient changes in pO_2_, are affected not only by oxygen supply (perfusion) but also by a variety of factors, including hemoglobin saturation and vascular architecture. Recently, Gaustad et al. showed that the Ktrans parameter derived from DCE-MRI compartmental analysis data is associated with tumor hypoxia in xenograft models of cervical carcinoma, melanoma, and pancreas (190). We present in Figure 4 an example of multimodality imaging of a patient with head and neck cancer.

Although widely used, the demonstration of perfusion variations is complex because many factors can influence it, such as the histological type of the tumor, the vascular architecture or the tumor size. No clinical study has currently shown a direct correlation between DCE-MRI and tumor hypoxia. The perfusion study by DCE-MRI seems to reflect indirect estimates of tumor hypoxia and in some circumstances do not correspond to hypoxia (189). The knowledge about tumor vasculature structure (DCE-MRI) and function ([^18^F]-FMISO PET) may be useful in newly diagnosed glioblastoma to provide prognosis information (191).

#### 4.4.2. Free oxygen mapping

Properly oxygenated tissues show almost complete saturation of hemoglobin molecules with O_2_. Diffusion of oxygen is lower in some tissues of the hypoxic tumor due to distance or vessel architecture. Oxygen has paramagnetic properties. As a result, dissolved oxygen in blood and plasma decreases the T1 value (16, 192). Under hyperoxic conditions, excess O_2_ is transported via the blood plasma into the tissues when perfusion is sufficient. The excess oxygen delivered remains dissolved in the blood plasma and tissue interstitial fluid, where it decreases the longitudinal relaxation time T1. It is then possible to observe this decrease at the voxel level by acquisition before and then after inhalation of hyperoxic gas. Theoretically, this decrease is proportional to the concentration of O_2_ dissolved in the tissues. It is significant in normoxic tissues because of a high concentration of dissolved O_2_ in these tissues, whereas a small or no decrease in T1 is demonstrated in hypoxic tissues, because of consumption of oxygen supply. However, the signal change is very small and this contrast mechanism has therefore only recently been exploited due to technological improvements in MRI imagers and developments in methods of analysis of the resulting images. Oxygen-enhanced MRI (OE-MRI) exploits the sensitivity of T1 to paramagnetic molecular oxygen dissolved in blood plasma or interstitial tissue using a hyperoxic gas test (193, 194).

Pre-clinical studies have shown significant differences in T1 decrease between normoxic and hypoxic tissue in a variety of tumor models (170, 195, 196). A human study has shown that the signal change is up to 20% and can be reliably measured in a range of normal, well-vascularized tissues with good tolerance of the technique (197). The small decreases in T1 were correlated with the hypoxic fraction and intra-tumor vessel density (198). Remmele et al. in 4 patients with brain tumor and Zhou et al. in 10 patients with prostate cancer have shown that these OE-MRI techniques can quantify and spatially map tumor hypoxia (199, 200).

However, almost all of the data are from studies in animal tumor models and further research in humans is needed (201). It seems necessary to investigate the potential value of this technique in clinical applications for the identification of tumor hypoxia (202). Moreover, excess O_2_ is also related to the transport capacity of blood vessels and the degree of perfusion may vary over time or during a treatment. The study of O_2_ variations, under hyperoxic conditions, should be associated with the study of perfusion to differentiate normoxic and hypoxic tissues.

In a preclinical study, O’Connor et al. demonstrated different levels of intratumoral hypoxia by analyzing the oxygen fraction in perfused tumor regions using a combined analysis with DCE-MRI rather than using OE-MRI in isolation (193). This approach appears to achieve better performance than using only one of the MRI techniques. Salem et al. confirmed these results in 23 patients with non-small cell lung cancer (NSCLC) where OE-MRI, in combination with perfusion assessment, identified and mapped regional differences between hypoxic and normoxic tumors with IHC validation as well as the change in tumor hypoxia induced by radiotherapy treatment (203). But further work to qualify this combined approach needs to be initiated for use in subsequent larger clinical trials.

## 5. Discussion

Detection of hypoxic territories is important for predicting resistance to cancer treatments, whether chemotherapy or radiotherapy.

Concerning more specifically radiotherapy, the determination of tumor hypoxic territories depends on a thresholding which is often by consensus 1.4–1.6 TBR for FMISO. This threshold is quite low, as it is a low contrast imaging. This can cause difficulties in the interpretation of PET images and requires nuclear physicians trained in reading this type of image. This is why other PET tracers have been developed such as FAZA (more hydrophilic), HX4 (more hydrophilic), or EF5 (more lipophilic) for example. These tracers have the same structure as the nitroimidazole tracers and provide information similar to that of FMISO, even if the biodistribution in the body is slightly different. Labeling with gallium-68 of a nitroimidazole does not seem to be better than fluorine-18, however this technique has the advantage of having a localized production near the nuclear medicine department. Cu-ATSM has the advantage of better TBR but there seems to be differences in binding in hypoxic areas compared to FMISO or FAZA. These differences may be explained by differences in the tracer retention mechanism.

Girentuximab is promising, with applications in PET imaging when labeled with zirconium-89 or in therapy. It allows the detection of cells overexpressing CA-IX and is well tolerated. However, to our knowledge, there is no clinical study comparing it to FMISO (or another nitroimidazole tracer).

MRI is another way to determine hypoxic regions. Free water movement can be studied as a reflection of tumor or tissue perfusion. The BOLD effect can be used to determine tissue oxygen levels and thus predict hypoxic tissue. DCE-MRI is a technique to know the distribution of hypoxic tissues, especially in tumors. The derived parameters (Ktrans, Vp, Ve) must however be carefully adjusted. Nevertheless, it is still difficult to determine the share of chronic hypoxia from transient hypoxia in MRI.

The alternative use of these new advanced MRI procedures (BOLD, perfusion, diffusion, etc.) able to assess tissue hypoxia could constitute another extremely interesting possibility, as it would avoid the use of hypoxia PET radiotracers (such as [^18^F]-FMISO or [^18^F]-FAZA), which are difficult to obtain from specialized manufacturers or must be locally produced, costly and time-consuming, and whose use in routine practice remains problematic because of the lack of thorough clinical validation and institutional approval.

The imaging techniques can be compared. PET imaging techniques have a low spatial resolution (3–5 mm), need the injection of radioisotopes and sometimes the tracers are not easily available (43). [^18^F]-nitroimidazole compounds provide a low contrast imaging. Because of that, this imaging method is sensitive to the threshold value to determine the hypoxic volume. However, a consensus with a maximal tumor-to-muscle ratio with 1.6 threshold seems robust enough to be used in multicentric studies (68). MRI techniques have a high spatial resolution and may necessitate the injection of contrast agent. The BOLD sequence are very sensitive to movement and to blood flow (16, 176), the OE-MRI sequence is sensitive to free oxygen in blood and tissue and may be able to identify hypoxic areas, DCE-MRI sequence provides knowledge on tumor vasculature and thus an indirect estimate of tumor hypoxia correlated with histological section stained with pimonidazole (165, 204). Voxel-wise fusion of ADC and Ktrans is an approach to generate hypoxia images in cervical cancer that can be implemented without requiring a true hypoxia measure (205). Nevertheless, hypoxia may be dynamic and transient (acute hypoxia) and images can be blurred or variable over time.

Hillestad et al. showed that a combination of Ktrans and Ve (rather than Ktrans and Ve alone) can be used to predict tumor hypoxic fraction and the hypoxia level in cervical cancer (204). These DCE-MRI parameters from the Tofts’ pharmacokinetics model reflected supply and consumption of oxygen and could be combined to generate hypoxia images of tumors in patient (183). This technique may be implemented in clinical routine. It was compared preclinically pO_2_ electron paramagnetic resonance imaging (EPRI), [^18^F]-FMISO uptake and DCE-MR imaging where correlations were observed. EPRI could be clinically used to better define tumor hypoxia (206). Recently a clinical study involving 17 patients with malignant gliomas evaluated MRI and [^18^F]-FAZA PET. A correlation was found with histological features (207). [^18^F]-FMISO PET/MRI could be used for imaging high-grade gliomas, especially glioblastoma multiforme, where the presence of [^18^F]-FMISO identifies hypoxic adaptation and helps delineate the spread of high-grade tumor tissue, even without gadolinium contrast enhancement on MRI (208).

## 6. Future perspectives

Tumor hypoxia is a complex phenomenon that would be useful to identify before and during treatment, as it is associated with a poor prognosis and clinical response. Identifying patients likely to benefit from a treatment adapted to the aggressiveness of their pathology is a major challenge of personalized medicine.

To visualize tumor hypoxia, the non-invasive methods offered by PET/CT and MRI are numerous and complex to implement. In addition, they provide partial images of hypoxia, which are generally difficult to interpret.

Clinical strategies for hypoxia imaging are dominated by PET/CT imaging (209), but no tracer is currently optimal for identifying tumor hypoxia. PET imaging with ^18^F-FDG is generally available and already provides metabolic information. Nevertheless, this type of exploration must be completed by other tracers more specific of hypoxia. Ideally, a PET tracer of hypoxia should reflect cellular rather than vascular pO_2_, at clinically relevant oxygen concentrations and only in viable cells, have uniform and rapid entry into cells, rapid clearance of normoxic cells, a high perfusion-independent hypoxic tumor-to-normoxic tissue ratio, and resistance to non-hypoxia dependent metabolism (189).

Many PET tracers have been proposed in the literature, but none of the radiotracers that have been used in clinical studies to date are perfect, and the development of more tumor hypoxia-specific radiotracers is still ongoing. Each radiotracer has advantages and dis-advantages and each can only be used in a relatively limited area. Despite this, ^18^F-FMISO remains the gold standard radiotracer for hypoxia.

On the other hand, MRI has some advantages over PET in terms of availability, ability to perform repeated measurements, greater sensitivity to different levels of hypoxia, and better spatial resolution to quantify tumor hypoxia locally. The literature review shows that there are several techniques: dynamic contrast-enhanced MRI (DCE-MRI), BOLD imaging, quantitative MRI (qMRI), water diffusion study, and OE-MRI. However, the relationship between the signal obtained in MRI and pO2 is complex.

## 7. Conclusions

We have focused in this review on the imaging of hypoxia by use of different ways, such as redox, metabolism, CA-IX expression, MRI techniques.

Hypoxia imaging by nitroimidazole compounds remains a low contrast imaging. The reference PET tracer is always [^18^F]-FMISO, but other tracers like [^18^F]-FAZA or [^18^F]-HX4 can be used. [64Cu]-ATSM is interesting but needs more clinical validation. MRI imaging is an emerging field in hypoxia. DCE-MRI might be used as a surrogate marker of hypoxic territories without injection of radioactive tracers.

## Author contributions

PB and PG: data collection, writing, analyzing, and setting up manuscript. ST, IG, PD, and PV: analysis and review. All authors contributed to the article and approved the submitted version.
